# Family structure and women’s mental well-being: how family stressors explain mental health inequalities between lone and partnered mothers

**DOI:** 10.3389/fsoc.2024.1498987

**Published:** 2024-12-04

**Authors:** Cadhla McDonnell, Pablo Gracia

**Affiliations:** ^1^Trinity College Dublin, Dublin, Ireland; ^2^Universitat Autònoma de Barcelona, Cerdanyola del Vallès, Spain; ^3^Centre d’Estudis Demogràfics, CED-CERCA, Cerdanyola del Vallès, Spain

**Keywords:** family structure, mothers’ mental well-being, maternal mental health, lone motherhood, stressors

## Abstract

Lone mothers have been found to report lower average mental health than partnered mothers. Following the ‘stress process model’, disparities in women’s mental health by family structure could be explained by lone mothers’ higher exposure to multiple forms of stressors, compared to partnered mothers. Yet, this hypothesis has not been tested in previous studies. This study analysed four waves of longitudinal data from the *Growing Up in Ireland* study, spanning between the year when women gave birth (2008) to 9 years later (2017) (*N* = 5,654 women), to examine how family stressors (i.e., financial strain, caregiving strain, work-related strain, and parental conflict) influence mothers’ depressive symptoms by family structure. Analyses applied random-effects models and Karlson-Holm-Breen (KHB) decomposition techniques, combined with different model specifications as robustness checks (i.e., fixed-effects). Results indicate that: (1) net of sociodemographic factors, lone mothers experience higher levels of depressive symptoms than partnered mothers, with additional analyses confirming that transitioning from partnered to lone mother is associated with higher depressive symptoms, and from lone to partnered mother with reduced depressive symptoms; (2) although 41% of the observed statistical association between family structure and mothers’ depressive symptoms is direct, a larger 59% of this mental health gap is mediated by inequalities between lone and partnered mothers in their exposure to family stressors; and (3) the largest share of the observed mediation by family stressors is explained by lone mothers’ higher risks of current and past caregiving strain and parental conflict, but also by their current higher financial strain. Overall, this study suggests that lone mothers’ lower mental health, compared to partnered mothers, is largely explained by disparities in exposure to family stressors, pointing to how accumulated caregiving and parental stressors, as well as poverty risks, are key explanatory factors behind the mental well-being disadvantage that lone mothers face.

## 1 Introduction

The presence of one-parent families in industrialised societies started to increase sharply around the 1960s, driven by major societal shifts in family values and attitudes linked to the ‘Second Demographic Transition’ (Lesthaeghe, 2010). Today, despite significant country variations and changing trends, one out of five families with children in OECD countries are headed by a lone mother (OECD, 2020). Previous studies found that lone mothers are at increased risk of suffering poor mental health such as depression and anxiety (Colton et al., 2015; Liang et al., 2019; Targosz et al., 2003). Lone mothers were also found to report lower levels of life satisfaction, less happiness, and more physical health problems, compared to partnered mothers (Baranowska-Rataj et al., 2014; Cetre et al., 2016; McDonnell et al., 2019; Umberson et al., 2010). Considering the well-established disadvantage that lone mothers experience in their mental health, understanding what drives wellbeing disparities between lone and partnered mothers is critical to research on families and health.

In this study, we examine the role of family stressors in shaping mental health disparities between lone and partnered mothers. Our study specifically seeks to answer the following research question: to what extent do family stressors, including financial strain, caregiving strain, work-related strain, and parental conflict, contribute to explain lone mothers’ higher risks of experiencing mental health problems (i.e., depressive symptoms), compared to partnered mothers?

Drawing on the ‘stress process model’ (Pearlin et al., 1981; Pearlin, 1999), we argue that mental health gaps across women with different family structures are explained by a higher exposure to multiple stressors among lone mothers, relative to partnered mothers. Some cross-sectional studies seem to indicate that higher exposure to stressors (e.g., financial hardship, caregiving strain) among lone mothers is associated with their poorer mental health relative to partnered mothers (Avison et al., 2007; Cairney et al., 2003). Yet, cross-sectional designs, as opposed to longitudinal designs, present empirical limitations. First, unlike longitudinal designs, cross-sectional designs impede to differentiate between cumulative exposure to stressors (i.e., repeatedly over time) and a single time exposure (i.e., in a periodic event). Second, as opposed to longitudinal designs, cross-sectional designs restrict our ability to account for the role of both current and past conditions when identifying causal mechanisms linking maternal stress exposure to mental health outcomes (e.g., Slopen et al., 2018; Turner and Lloyd, 1995).

To our knowledge, only one study has longitudinally investigated how stress-related mechanisms explain mental health disparities between lone and partnered mothers. Using two waves of longitudinal survey data and employing random-effects models with Canadian data collected from 1989 to 1991, Avison et al. (2007) found that the association between partnership status and maternal psychological distress is partly driven by different stress exposure (e.g., caregiving and financial stress) between lone and partnered mothers. While this study adds to the literature, further research is needed. First, more recent evidence focusing on national contexts that were not previously studied is needed to expand our knowledge of the mechanisms driving gaps in mothers’ mental health by family structure. This is particularly relevant considering that one-parent and two-parent families differ in their demographic characteristics across national contexts and cohorts (Bernardi and Mortelmans, 2018; Chzhen and Bradshaw, 2012; Nieuwenhuis, 2020; Nieuwenhuis and Maldonado, 2018). Second, a formalised mediation approach that allows to show longitudinally how different stressors at multiple time points contribute to explain mental health disparities between lone and partnered mothers (e.g., financial strain versus caregiving strain) has not been yet applied. Third, existing longitudinal evidence is based on two waves of observation in a short-term period (Avison et al., 2007). Our study addresses these important gaps, seeking to contribute to our understanding of the causal mechanisms driving differences in mothers’ mental health by family structure.

The present study adds to the family and health literatures by examining systematically how exposure to different family stressors over time links to disparities in depressive symptoms between lone and partnered mothers. We do so by analysing high-quality, longitudinal data for a large population-based sample of mothers in Ireland who were interviewed at four different time points between 2008 (the year in which they gave birth) and 2017 (9 years after childbirth). Specifically, we formally test the relative role that various forms of past and current family stressors play in shaping mental gaps depending on women’s family structure, namely ‘financial strain’, ‘caregiving strain’, ‘work-related strain’, and ‘parental conflict’. In doing so, our study brings a novel investigation of precise mechanisms of exposure to stressors that can lead to differences in mothers’ mental health across different family structures.

## 2 Background

### 2.1 The Irish case

To study the links between family structure and parental well-being in Ireland, it is important to highlight the structural context of policies and norms around families and care work in this country. The literature on the Irish welfare, family and gender regimes indicates a relatively genuine hybrid or mixed model in the context of high-income industrialised countries. Ireland presents clear elements of a neo-liberal market-oriented social policy framework, characterised by mean-tested policies that guarantee residual universal public support oriented toward families (Daly, 2018). This limited system of service provision in Ireland has been traditionally organised in the form of a partnership between the state and the voluntary sector, with the Catholic church playing a pivotal role in shaping traditionally conservative public policies and family relations (Daly, 2018). The Irish system presents a high incidence of the 1.5 couple model, with 31% of female part-time employment, compared to an average of 22% in OECD countries (OECD, 2024), coupled with levels of in-work poverty and part-time job incidence among Irish lone-parent families around the EU average (Nieuwenhuis, 2020). Ireland additionally presents marked gendered role expectations and attitudes toward the family, as illustrated in research showing clear gender gaps in the division of domestic labour, with women doing a much larger share of domestic work (McGinnity and Russell, 2008). With regards to childcare provision, the Irish system of early childhood education is expensive and highly commodified, which has been linked to higher family pressures among families with higher risks of economic vulnerability, such as low-income and one-parent families (Pietropoli and Gracia, 2022). Overall, the policy and normative contexts of families and gender relations in Ireland need to be considered to understand the potential disadvantages that lone mothers may face in this country, compared to partnered mothers.

Despite the persistence of traditional and conservative elements shaping family relations, Ireland has experienced major demographic changes in recent decades, many of them associated with greater plurality of family forms and gender attitudes (Canavan, 2012). The Irish context presents an interesting combination of traditional conservative family relations with rapid recent demographic transformations linked to the ‘Second Demographic Transition’. Today, rates of lone motherhood in Ireland are similar to those observed in many other high-income Western European countries (OECD, 2020), even if divorce became legal in Ireland only in 1996, much later than many other high-income industrialised countries.

To date, research on the impact of family structure on various spheres of life in Ireland is scarce, limited to specific snapshots addressing primary school children (Hannan, 2018). The literature situates Ireland as notable for the large ‘happiness penalty’ experienced by parents, and mothers particularly. A study investigating how stressors and resources shape wellbeing in 22 high-income countries found that Ireland had the second largest drop in happiness associated with parenthood (Glass et al., 2016), which may reflect greater exposure to parenting stressors in Ireland due to limited government family support. In previous cross-sectional studies with Irish data, lone mothers reported much higher depressive symptoms than married mothers (Fahey et al., 2012; McKeown et al., 2003). However, there is no longitudinal research on maternal mental health disparities by family structure in the Irish context. Therefore, we not only add to the literature by systematically studying the mediating role of exposure to stressors in shaping mental health disparities between lone and partnered mothers, but we also do it for a national context (i.e., Ireland) for which little research has been conducted on this topic.

### 2.2 The role of family structure in family stressors and mothers’ mental health

Our theoretical framework draws strongly on the ‘*stress process model*’, which was originally developed to study the socially patterned distribution of mental health outcomes (Pearlin et al., 1981; Pearlin, 1999), and has been recently applied to study parents’ mental health (Costanzo et al., 2024; Nomaguchi and Milkie, 2017; Nomaguchi and Milkie, 2020; Manuel et al., 2012). Under this model, stress is conceptualised as a process that combines three components: (i) ‘sources of stress’ (i.e., stressors), (ii) ‘moderating resources’ (i.e., buffers), and (iii) ‘outcomes of stress’ (i.e., mental health outcomes) (Pearlin et al., 1981; Pearlin, 1999). Stressors include stressful life events, traumas and ongoing strains, such as financial difficulties, work constraints, relationship issues and parenting conflicts (Turner et al., 1995; Wheaton, 1999). As part of the ‘stress process model’, exposure to stressors are hypothesised to have direct negative effects on wellbeing outcomes, such as depressive symptoms and anxiety (Pearlin et al., 1981; Pearlin et al., 1990; Thoits, 2010).

Within the ‘stress process model’, stress is conceived as a non-static, dynamic process that accumulates over time, where initial stressors can lead to secondary stressors that cause multiple cumulative processes of stress proliferation (Pearlin, 1999). For example, an unplanned pregnancy could lead to a multiplied proliferation of stressors such as financial strain, work interruptions or strained family relationships that would, in combination, cause a decline in a woman’s wellbeing. Whether caused by an accumulated exposure to multiple types of stressors or to a repeated exposure to the same ongoing stressor, individuals’ cumulative exposure to stressors over time has been found to be particularly detrimental for their mental health trajectories (Slopen et al., 2018; Turner and Lloyd, 1995; Turner et al., 1995; Thoits, 2010). Although the ‘stress process model’ has direct applications to study disparities in mothers’ mental health between lone and partnered mothers, this model has been omitted from existing literature on differences in mothers’ wellbeing by family structure. Specifically, the stress model has been largely omitted from the literature addressing. Our study fills in this gap by readapting the ‘stress process model’ to study the role of exposure to stressors in shaping mothers’ mental health gaps by family structure.

To date, very few studies have examined how stress exposure links to maternal mental health differences by family structure, with most evidence being based on cross-sectional data. A two-year study by Brown and Moran (1997) on a sample of 400 women with young children from North London in the UK investigated the incidence of maternal depression by partnership status, indicating that financial hardship, marital difficulties and widowhood are associated with lone mothers’ poorer mental health than partnered mothers. A cross-sectional analysis on depression among 3,000 mothers in Canada found that differences in chronic strain, stressful life events, and childhood adversity accounted for a third of the difference in depression between lone and partnered mothers (Cairney et al., 2003). Also using Canadian data, one study that combined cross-sectional and longitudinal methods to a sample of 1,000 mothers (Avison et al., 2007) found that differences in psychological distress between lone and partnered mothers were no longer statistically significant once background characteristics, chronic strain, and stressful life events were considered. Finally, using representative German data for a large sample of more than 11,000 women, Recksiedler et al. (2023) examined how stressors concerning mothers’ subjective, relational, and financial well-being accumulate and combine within subgroups of mothers with different types of family structure, and found that single mothers are disproportionately at risk of belonging to the most vulnerable group in terms of exposure to multiple stressors.

While the very few identified studies suggest that an unequal exposure to stressors may contribute to explain poorer mental health for lone mothers than for partnered mothers, existing literature presents shortcomings that motivate further research. First, most research on the associations between family structure and mothers’ mental health is cross-sectional and omits a test of mechanisms. This undermines our ability to draw conclusions on the conditions under which family structure is associated with disparities in maternal mental health. Second, the scarce longitudinal literature on this topic has overlooked a formal mediation analysis, not only to test the stress process model by examining direct and indirect associations between family structure and mothers’ mental health, but also to quantify the relative salience of different stressors in explaining mental health inequalities in mental health by mothers’ family structure. Our study addresses these knowledge gaps by situating the ‘stress process model’ within a dynamic framework that allows to test how differential exposure to family stressors shapes existing mental health gaps between lone and partnered mothers.

We specifically assess the role of four key family stressors—financial strain, caregiving strain, work-related strain, and parental conflict—in explaining how mothers’ mental health differs by family structure. Globally, we hypothesise that poorer mental health among lone mothers is driven by their higher exposure to stressors, compared to partnered mothers. The rationale to expect how each family stressor within our study shapes differences in mothers’ mental health by family structure is explained below, assuming that (1) lone mothers experience these stressors at higher rates than partnered mothers, and (2) each of these stressors is associated with maternal mental health problems.

#### 2.2.1 Financial strain

Previous research found that lone mothers experience high rates of financial strain, and also that financial strain negatively influences individuals’ mental health. Previous studies indicate that poverty and financial strain are substantially higher among lone mothers than among partnered mothers (Chzhen and Bradshaw, 2012). Furthermore, a substantial body of research has documented the impact of financial hardship on adults’ (and mothers’) mental health problems over the life course (Crosier et al., 2007; Dijkstra-Kersten et al., 2015; Kingston, 2013; Zimmerman and Katon, 2005). As a result, one may expect that past and current financial strain can contribute to poorer mental health outcomes among lone mothers than among partnered mothers.

#### 2.2.2 Caregiving strain

Increased stress related to the parenting role (i.e., caregiving strain) may contribute to worse mental health outcomes among lone mothers. Multiple studies found that mothers who parent without a co-resident partner report higher levels of caregiving strain, responsibilities and time demands (Avison et al., 2007; Cano and Gracia, 2022; Davies et al., 1997; Fahey et al., 2012). Caregiving strain has also been shown to have a positive association with mothers’ depressive symptoms in a variety of contexts (Huang et al., 2014; Weitlauf et al., 2014). Altogether, following previous literature, it is reasonable to expect that past and current caregiving strain contributes to explain the hypothesised lower levels of mental health for lone mothers than for partnered mothers.

#### 2.2.3 Work-related strain

Lone mothers have increased challenges in combining paid work with family responsibilities, and this may contribute to their mental health disadvantages. Lone mothers, also specifically in Ireland, are substantially less likely to engage in paid work than partnered mothers, in large part due to the high cost of childcare (Regan et al., 2018). Mothers who are not in paid employment have worse long-term mental and physical health outcomes compared to those who are in employment (Crowe and Butterworth, 2016; Whelan et al., 1991). Also, high levels of work–family conflict can lead to increased depressive symptoms among mothers (Frone et al., 1997; Goodman et al., 2009), and previous research found that work–family conflict harms lone mothers disproportionately (Dziak et al., 2010). Hence, there are reasons to expect that differential exposure to past and current work-related strain by family structure contributes to explain poorer mental health among lone mothers, compared to partnered mothers.

#### 2.2.4 Parental conflict

Lone mothers are more likely than partnered mothers to face family conflict, whereas lone mothers seem more prone to have experienced higher conflict with their children’s parents at some stage (Fagan and Palkovitz, 2011; Fahey et al., 2012). High levels of conflict with another parent in charge of the child was found to be associated with an increase in mothers’ depressive symptoms that persists over time (Barton et al., 2017; Paulson et al., 2011). Consequently, if parental conflict impacts lone mothers disproportionately, it is reasonable to expect that such different exposure to parental conflict by family structure contributes to explain lone mothers’ lower mental health levels, relative to partnered mothers.

## 3 Data and methods

### 3.1 Data and sample

We use data from the 2008 cohort of the *Growing up in Ireland* study (GUI), an ongoing study of Irish families with children. Families in the 2008 cohort of the GUI were randomly selected from those who had an infant born between December 2007 and June 2008 and registered on the Child Benefit Register. The longitudinal, nationally representative nature of the data makes it ideal for determining how family structure and stressors impact mental health among mothers over time. Interviews were conducted with primary caregivers at multiple time points. The GUI contains relevant sociodemographic data and information on various stressors and mental health outcomes for mothers. We use data from surveys collected in Year 1, Year 3, Year 5, and Year 9, namely 2008, 2011, 2013, and 2017/18, respectively. Our sample compares mothers’ mental health across two groups: (1) partnered mothers, and (2) lone mothers. To avoid the potentially confounding impact of partnership transition on stress and wellbeing in our mediation framework, and also to ensure that the decomposition analysis allows for a stable comparison of groups over time without facing constraints in statistical estimations, we limit our sample to mothers whose family structure remained stable over the observation years of our study. In our sampling, we kept all biological and adoptive mothers who were the primary caregiver of the study child, and for whom we had complete information on all study variables across the four waves of observation. Therefore, cases where there was attrition and/or cases with incomplete information in at least one of measure of study were excluded from our analyses, given that our longitudinal framework requires complete information for all variables of study across waves. This led us to reduce our sample from 8,488 women in the early wave to our total final sample containing complete information across all waves of study: a total of 5,654 women.

### 3.2 Variables

#### 3.2.1 Dependent variable

Our dependent variable is a score of depressive symptoms. Depression is a common form of psychological distress that is experienced by most people to some degree at some point in their lives. Depressive symptoms is considered to be a useful dependent variable for investigating how social and economic factors impact mental health (Pearlin et al., 1981). We use the self-report CES-D 8, a short form of the original 20-item Centre for Epidemiological Studies Depression Scale (CES-D) to assess mothers’ depressive feelings and behaviours during the previous week. Items from this scale include poor sleep, crying, feelings of fear, loneliness, sadness, failure, and depression. We used the raw CES-D score, allowing us to examine variations in psychological distress, even at subclinical levels. The resultant variable, *depressive symptoms*, which we treated as continuous, ranges from 0 to 24 with higher values indicating higher levels of psychological distress.

#### 3.2.2 Independent variable

Our main independent variable was *lone mother*, which is coded 0 for partnered mothers and 1 for lone mothers, based on the GUI household level data.

#### 3.2.3 Mediating variables

We examine four measures of stress exposure: (1) financial strain, (2) caregiving strain, (3) work-related strain, and (4) parental conflict. *Financial hardship* was measured using a survey item from quasi-cardinal scales that provide ordinal measures of agreement through six-point Likert scales derived from questions asking about the family’s ease or difficulty in making ends meet. This resulted in a six-point variable, where higher values indicated greater difficulty in making ends meet. *Caregiving strain* was measured using the six-item parental stressors subscale from the Parental Stress Scale (Berry and Jones, 1995). Respondents used a 5-point, Likert-style scale to report their agreement or disagreement with six validated statements on the impact of parenting on various dimensions of wellbeing, such as for example whether it was true that having children leaves little time or energy for non-child related activities and whether it is difficult to balance different responsibilities because of one’s children. Responses were averaged to produce a 5-point ordinal variable, where higher values indicate greater caregiving strain*. Work-related strain* was a categorical variable, using mothers’ reported work status and survey items from the GUI about work-family strain. These items asked respondents, in questions formulated as five-point Likert scales, to report how job responsibilities link to participation and enjoyment of home life, with questions asking for instance whether one’s job prevents from spending sufficient quality time with family members. We averaged these four survey items to create a measure of work-family strain. We then used this measure, along with mothers’ employment status, to specify a new, four-point categorical variable, including mother’s ‘non-employment’, and then three levels of work-family strain: ‘low’, ‘medium’, and ‘high’. *Parental conflict* was based on mothers’ reports of whether the study child experienced substantial conflict at home between the mother and the other parent, captured with a dummy variable adopting the code 0 to indicate no substantial parental conflict and the code 1 to indicate substantial parental conflict. Our measure of parental conflict was available in wave 3 and 4, but not in wave 1 and 2.

#### 3.2.4 Control variables

The disparity in mental health between lone and partnered mothers is partly due to the selective nature of lone parenthood (Cairney et al., 2003; Davies et al., 1997; McKeown et al., 2003; McLanahan, 2004). Both age and education are associated with depressive symptoms (Turner et al., 1995), and lone and partnered mothers in Ireland differ on average age and education levels (Hannan, 2018). Respondents’ *age* was measured in years. *Education* was a six-point scale indicating the highest level of education mothers had attained. Lone mothers in Ireland and elsewhere tend to enter into parenthood younger than partnered mothers (Harkness, 2018; Punch, 2006), and depressive symptoms are significantly associated with age at first birth (Carlson, 2011; Mirowsky and Ross, 2002). *Age at first pregnancy* was measured in years. *Irish born* was a dummy variable indicating whether the respondent was born in Ireland. *Early economic deprivation* was a six-point ordinal variable with higher values indicating greater financial hardship when the respondent was young, as previous research has reported associations between depressive symptoms and socioeconomic status or economic deprivation (Hoebel et al., 2017; Tracy et al., 2008). We accounted for the number and ages of the respondents’ children, which was found to be associated with depression (Fahey et al., 2012). *Family size* was a continuous variable measuring the number of children in the household, including respondents’ biological, adopted, and stepchildren. *Age of youngest child* was measured in years.

### 3.3 Empirical strategy

Our empirical strategy follows three steps. First, we report descriptive statistics for lone and partnered mothers to investigate how depressive symptoms, sociodemographic characteristics, and stressors vary by family structure at each year of observation. We present descriptive analyses with summary statistics for each variable, and then conduct statistical bivariate correlations to report whether differences between lone and partnered mothers were statistically significant for each measure of study, based on the obtained *p*-values.

Second, we conduct longitudinal analyses to examine how depressive symptoms are associated with family structure, net of multiple relevant factors. Random-effects models differ from fixed-effects models in allowing to estimate coefficients for time-invariant and time-variant measures by comparing differences over time across stable groups (Clark and Linzer, 2015). Additionally, random-effects are less sensitive to sampling variability than fixed-effects, while our study focuses on stable—time-invariant—measures of family structure in order to allow our longitudinal decomposition analysis to estimate past and current stressors within our mediation model framework (Clark and Linzer, 2015). This led us to opt for random-effects models, instead of fixed-effects models, in our main analyses. Despite the reasons behind our statistical choice, we must acknowledge that our restriction to mothers with a stable family structure throughout the window of observation of our study is (i) limiting the scope of our analyses to a larger population, and (ii) challenging our ability to control for unobserved heterogeneity through a fixed-effects model estimation (Allison, 2009) For this reason, we conducted additional analyses as robustness checks by exploiting the within-individual change (transitioning from partnered to lone mother, and vice versa) to further confirm that (a) similar patterns apply for a larger sample, and (b) results are consistent when controlling for unobserved heterogeneity with a more restricted model with time-varying measures only. Our main analyses with random-effects models follow four steps. In Model 1 we include only family structure as predictor of mothers’ depressive symptoms. In Model 2 we expand the model to include sociodemographic characteristics that may be related to both family structure and mental health outcomes. In Model 3 we further include three stressors: financial strain, caregiving strain, and work-related strain. Finally, in Model 4, we add the fourth mediating stressor: parental conflict. The last model includes data from the last two waves only, as information for parental conflict was available for Year 5 and Year 9 only, which explains why this variable was added separately in a last model.

Third, we conduct decomposition models to test how past and current stressors mediate differences in depressive symptoms by family structure. We run Karlson-Holm-Breen (KHB) path decomposition models (Karlson et al., 2012; Breen et al., 2021). In the KHB method, the coefficients from the reduced model (without mediators) are compared to those in the full model (with mediators) after accounting for residuals. The difference between the reduced and full models is the ‘indirect effect’ and the percentage of the effect that is mediated is calculated by means of dividing the indirect effect by the total effect across stable groups over the different time points include in the study. Empirical analyses were performed with Stata 16 by using the *khb* command, which applies to different types of outcome measures. Although the KHB method was originally developed for binary, logit and probit models, this empirical technique works for nonlinear probability models (ordered and multinomial), as well as for linear regressions (see Breen et al., 2021). Our KHB analyses were weighted to account for attrition, using the weights supplied by the GUI across all four waves of study.

## 4 Results

### 4.1 Descriptive analyses

Table 1 presents weighted proportions or means and standard deviations for all our study variables. Table 1 further indicates whether there are statistically significant differences by family structure, based on bivariate analyses showing if differences between lone and partnered mothers for each measure of study are statistically significant.

**Table 1:** Weighted means (M) and proportions (Prop) and standard deviations (s.d.) of variables for lone mothers and partnered mothers by survey year.

	Year 1				Year 3					Year 5					Year 9				
	Lone mothers		Partnered mothers		Lone mothers		Partnered mothers			Lone mothers		Partnered mothers			Lone mothers		Partnered mothers		
*Variables*	*M/prop.*	*s.d.*	*M/prop.*		*M/prop.*	*s.d.*	*M/prop.*		*s.d.*	*M/prop.*	*s.d.*	*M/prop.*		*s.d.*	*M/prop.*	*s.d.*	*M/prop.*		*s.d.*
CES-D score	4.73	3.67	2.14	***	3.75	3.02	2.10	***	3.40	3.72	3.43	2.02	***	3.30	4.16	3.50	2.00	***	3.04
Age at first pregnancy (measured at Year 1)	23.70	4.58	27.90	***															
Early economic deprivation (measured at Year 1)	3.83	0.88	3.58	**															
Born in Ireland (measured at Year 1)	0.79		0.83																
Age	28.11	4.64	32.65	***	30.33	4.67	34.95	***	4.83	32.45	4.69	37.07	***	4.82	36.82	4.63	41.34	***	4.82
Number of children in household	1.55	0.52	2.00	***	1.67	0.60	2.38	***	1.02	1.72	0.62	2.64	***	1.00	1.96	0.77	3.80	***	1.18
Age of youngest child	0	0	0		2.71	0.56	2.09	***	1.28	4.46	0.92	3.22	***	1.89	7.44	1.96	6.43	***	2.72
Educational attainment																			
Less than secondary	0.37		0.14	***	0.32		0.11	***		0.27		0.09	***		0.24		0.09	***	
Secondary	0.35		0.23	**	0.27		0.17	**		0.25		0.14	**		0.22		0.13	**	
Some post-secondary	0.20		0.30	***	0.31		0.36			0.37		0.42			0.40		0.42		
Degree level	0.03		0.10	***	0.04		0.09	***		0.05		0.10	**		0.06		0.10	*	
Some postgraduate	0.05		0.23	***	0.06		0.27	***		0.05		0.25	***		0.08		0.26	***	
Financial strain (1–6)	4.04	0.76	3.24	***	4.37	0.80	3.63	***	1.14	4.39	0.82	3.79	***	1.12	3.91	0.83	3.22	***	1.08
Caregiving strain (1–5)	2.61	0.57	2.38	***	2.39	0.60	2.00	***	0.68	2.26	0.53	1.90	***	0.68	2.26	0.54	2.19		0.75
Work-related strain																			
Low work-family strain	0.13		0.18	^†^	0.12		0.17	^†^		0.09		0.16	**		0.18		0.22		
Medium work-family strain	0.08		0.13	**	0.07		0.14	**		0.08		0.13	**		0.17		0.17		
High work-family strain	0.14		0.15		0.14		0.19	*		0.15		0.25	**		0.21		0.25		
Non-employment	0.66		0.53	**	0.67		0.51	***		0.68		0.46	***		0.44		0.35	*	
Parental conflict										0.19		0.03	***		0.16		0.03	***	
*N*	264		5,390		264		5,390			264		5,390			264		5,390		

^†^*p* < 0.1, *p < 0.05, **p < 0.01, ***p < 0.001.

Statistical analyses were based on bivariate correlations for each measure of study to report whether differences between lone and partnered mothers were statistically significant, based on the p-values from the statistical correlations. The measure number of children in the household was calculated differently for Year 9 round compared to earlier rounds due to changes in the data released by the CSO. For Year 3 and Year 5, the number of children in the household was calculated to include all full-, half-, and step-siblings of the study child using the information in the household grid. For Year 9, it was calculated using derived variables from the CSO on the number of siblings older and younger than the study child living in the household. Age was measured in full years in the GUI household grid, hence at Year 1 the youngest child in all households was under 1.

Overall, respondents reported low to moderate levels of depressive symptoms in all years. Yet, there were clear differences in maternal depressive symptoms by family structure. On a scale of 0 to 24, lone mothers scored 4.73 at Year 1, 3.75 at Year 3, 3.72 at Year 5, and 4.16 at Year 9. Partnered mothers reported fewer depressive symptoms: 2.14 at Year 1, 2.10 at Year 3, 2.02 at Year 5, and 2.00 at Year 9. On average, lone mothers reported about twice as many depressive symptoms as partnered mothers, with the ratio of lone mothers’ to partnered mothers’ responses ranging from 2.21 at Year 1 to 1.84 at Year 5. These differences in depressive symptoms by family structure were statistically significant at all waves of study (*p* < 0.001).

Table 1 also shows that lone and partnered mothers differed substantially in their sociodemographic characteristics and in their exposure to different types of stressors. Most of these differences were statistically significant at the 95% confidence level or higher (*p* < 0.05), but we note it if exceptionally these differences were not statistically significant. Lone mothers reported younger age of first pregnancy and higher levels of early economic deprivation. At all survey years, lone mothers were younger, had lower levels of education and fewer children in the household. Lone mothers also reported older ages for their youngest children in Years 3, 5, and 9. There were differences between lone and partnered mothers’ exposure to all stressors in our analysis. At all survey years lone mothers reported more financial strain. Lone mothers also reported more caregiving strain at Years 1, 3, and 5 than partnered mothers. At Year 9, the raw differences in caregiving strain by family structure were smaller and not statistically significant. In all survey years, non-employment was more frequent among lone than among partnered mothers. At Years 1 and 3 partnered mothers reported experiencing medium levels of work-family strain more frequently, while at Year 5 partnered mothers reported experiencing low, medium, or high levels of work-family strain to a higher extent. Also, lone mothers reported that their child had witnessed parental conflict at higher rates than partnered mothers. These clear differences in background characteristics between lone and partnered mothers further motivate our multivariate longitudinal analyses.

### 4.2 Random-effects analyses

Table 2 presents the results of random-effects models on maternal depressive symptoms through four different models. In Model 1 of Table 2, when there are no other covariates, being a lone mother is associated with higher depressive symptoms (b = 1.86; *p* < 0.001).

**Table 2:** Random effects models predicting depressive symptoms using various covariates.

	Model 1			Model 2			Model 3			Model 4		
*Variables*	*Coeff.*		*S.E.*	*Coeff.*		*S.E.*	*Coeff.*		*S.E.*	*Coeff.*		*S.E.*
Lone mother	1.86	***	0.15	1.52	***	0.15	1.00	***	0.14	0.46	**	0.16
Age at first pregnancy				−0.03	**	0.01	−0.04	***	0.01	−0.05	***	0.01
Early economic deprivation				0.17	***	0.03	0.09	***	0.02	0.08	**	0.03
Irish born				−0.20	*	0.08	0.03		0.07	0.02		0.08
Respondent age				0.00		0.01	0.02	^†^	0.01	0.03	**	0.01
Number of children at home				−0.04		0.03	−0.13	***	0.03	−0.17	***	0.03
Age of youngest child				−0.01		0.01	0.03	**	0.01	0.02		0.01
Education^a^												
Secondary				−0.10		0.13	−0.05		0.12	−0.21		0.17
Some post-secondary				−0.31	**	0.12	−0.18	^†^	0.11	−0.28	^†^	0.15
BA				−0.25	^†^	0.13	−0.12		0.12	−0.30	^†^	0.17
Some post graduate				−0.50	***	0.13	−0.30	*	0.12	−0.42	**	0.16
Financial strain							0.25	***	0.02	0.28	***	0.03
Caregiving strain							0.96	***	0.03	1.11	***	0.04
Work-related strain^b^												
Medium w-f strain							0.07		0.06	0.14	^†^	0.08
High w-f strain							0.27	***	0.06	0.34	***	0.08
Not in paid employment							0.51	***	0.05	0.62	***	0.08
Parental conflict										1.23	***	0.13
Constant	0.08		0.16	1.25	***	0.32	−1.57	***	0.31	−1.48	***	0.39
Number of observations	22,616			22,616			22,616			11,308		
Number of respondents	5,654			5,654			5,654			5,654		
Prob > *X^2^*	0.000			0.000			0.000			0.000		

a Reference category is less than secondary education.

b Reference category is low work-family strain.

^†^*p* < 0.1, *p < 0.05, **p < 0.01, ***p < 0.001.

Model 4 contains the last two waves only, as information for parental conflict was only available for Year 5 and Year 9.

In Model 2 (Table 2), with sociodemographic characteristics included along with family structure, being a lone mother is still associated with higher depressive symptoms (b = 1.52; *p* < 0.001), with lone mothers’ CESD scores almost 1.5 points higher than those of partnered mothers when sociodemographic characteristics are accounted for. Higher levels of early economic deprivation and younger age at first pregnancy are both positively associated with depressive symptoms (b = 0.17; *p* < 0.001). We also observe that Irish birth is associated with fewer depressive symptoms (b = 0.20; *p* < 0.05). Finally, higher levels of education are generally negatively associated with depressive symptoms, with differences between postgraduate education and qualifications under secondary education being particularly pronounced (b = −0.50; *p* < 0.001).

In Model 3 (Table 2), which adds measures of financial strain, caregiving strain, and work-related strain alongside sociodemographic characteristics, we observe that a statistically significant difference in depressive symptoms between lone and partnered mothers remains (b = 1.00; *p* < 0.001), although the size of the difference is reduced by over a third, compared to Model 2. Higher levels of early economic deprivation and lower age at first pregnancy are still associated with increased depressive symptoms (b = 0.09; *p* < 0.001), though the size of the coefficient for early economic deprivation is substantially reduced. Irish birth is no longer associated with depressive symptoms in Model 3, and respondent age becomes positively associated with depressive symptoms (b = 0.03; *p* < 0.05), while having more children in the household is associated with fewer depressive symptoms (b = −0.13; *p* < 0.001) and an older age of the youngest child is associated with more depressive symptoms (b = 0.03; *p* < 0.05), and the coefficient for post-graduate education remains as statistically significant (b = −0.30; *p* < 0.05). Regarding the measures of stressors, financial strain, caregiving strain, and work-related strain are all associated with depressive symptoms. A one-point increase in financial strain is associated with an increase of 0.25 points in respondent CESD score (b = 0.25; *p* < 0.001), whereas a one-point increase in caregiving strain is associated with an increase of 0.96 points in respondent CESD score (*p* < 0.001). Both high maternal work-family strain (b = 0.27; *p* < 0.001) and not being in paid employment (b = 0.51; *p* < 0.001) are associated with higher maternal depressive symptoms, compared to mothers’ low work-family strain.

Finally, Model 4 (Table 2) adds measures for parental conflict along with other stress variables and sociodemographic characteristics. Because parental conflict was only measured at Years 5 and 9, this model uses data from these two waves only. We observe that a significant difference in depressive symptoms remains between lone and partnered mothers, though the size and significance of the coefficient is reduced compared to Models 1, 2, and 3. The associations between sociodemographic variables and mothers’ depressive symptoms are largely unchanged from Model 3, with the exception that there is no longer a statistically significant effect of the age of the youngest child on mothers’ depressive symptoms. The associations between financial strain, caregiving strain, and work-related strain remain similar to those in Model 3. Yet, we observe that parental conflict has a strong positive association with mothers’ depressive symptoms. Reporting that a child has experienced parental conflict is associated with an increase of almost 1.5 points in mothers’ CESD score (*p* < 0.001). In additional analyses as robustness checks (not shown), we ran Model 3 with observations from the last two waves only. These results were substantively similar to those from the original Model 3 with observations from all the waves, offering conclusive evidence that parental conflict is an important stressor regardless of sample specifications.

### 4.3 Mediation analyses: KHB decomposition models

Tables 3, 4 present the KHB decomposition analyses of the effect of family structure on mothers’ depressive symptoms at Year 9. In Table 3, we present the estimated total, direct, and indirect effects of family structure on depressive symptoms and the combined proportion that is mediated by all stressors in the model. In Table 4, we report the contribution that each individual stressor adds to the mediation, including the proportion of the indirect and total effect accounted for by each mediator.

**Table 3:** Decomposition of the effect of family structure on mothers’ depressive symptoms at Year 9 using the KHB method (*n* = 5,654).

	*Coeff.*		*S.E.*
Total effect	1.59	***	0.36
Direct effect	0.66	^†^	0.37
Indirect effect	0.93	***	0.17
Percent mediated			58%
*n=*	*5,654*		

^†^*p* < 0.1, ***p < 0.001.

**Table 4:** Components of difference from the decomposition of the effect of family structure on mothers’ depressive symptoms at Year 9 using the KHB method (*n* = 5,654).

Z- variable	*Coeff.*	*S.E.*	% indirect effect	% total effect
Year 9 mediators				
Financial strain	0.16	0.06	17%	10%
Caregiving strain	0.12	0.06	13%	8%
Non-employment^a^	0.09	0.04	10%	6%
Parental conflict	0.20	0.07	21%	12%
Year 5 mediators				
Financial strain	0.01	0.03	2%	1%
Caregiving strain	0.10	0.05	11%	6%
Non-employment^a^	−0.04	0.03	−4%	−3%
Parental conflict	0.15	0.07	16%	9%
Year 3 mediators				
Caregiving strain	0.04	0.03	4%	3%
Non-employment^a^	0.02	0.02	2%	1%
Year 1 mediators				
Financial strain	0.02	0.03	2%	1%
Caregiving strain	0.05	0.03	5%	3%
Medium work-family strain^a^	−0.02	0.01	−2%	−1%
Non-employment^a^	0.01	0.02	2%	1%

a Reference category is employed with low work-family strain.

Mediators that contributed to the indirect effect by less than 1% are not shown. Full results available upon request.

The KHB decomposition model presented in Table 3 was specified to include all stressors from Year 1 through Year 9. The results indicate that the total effect (*p* < 0.001) and the indirect effect (*p* < 0.001) of family structure on depressive symptoms was statistically significant at Year 9. By contrast, the direct effect shows there is no statistically significant difference in depressive symptoms between lone and partnered mothers. Whereas 41% of the difference in depressive symptoms between lone and partnered mothers remains unexplained in this model, exposure to stressors accounted for 59% of the effect of being a lone parent on depressive symptoms. This indicates that the effect of family structure on mothers’ depressive symptoms is substantially mediated by both current and past exposure to stressors.

**Figure 1:**
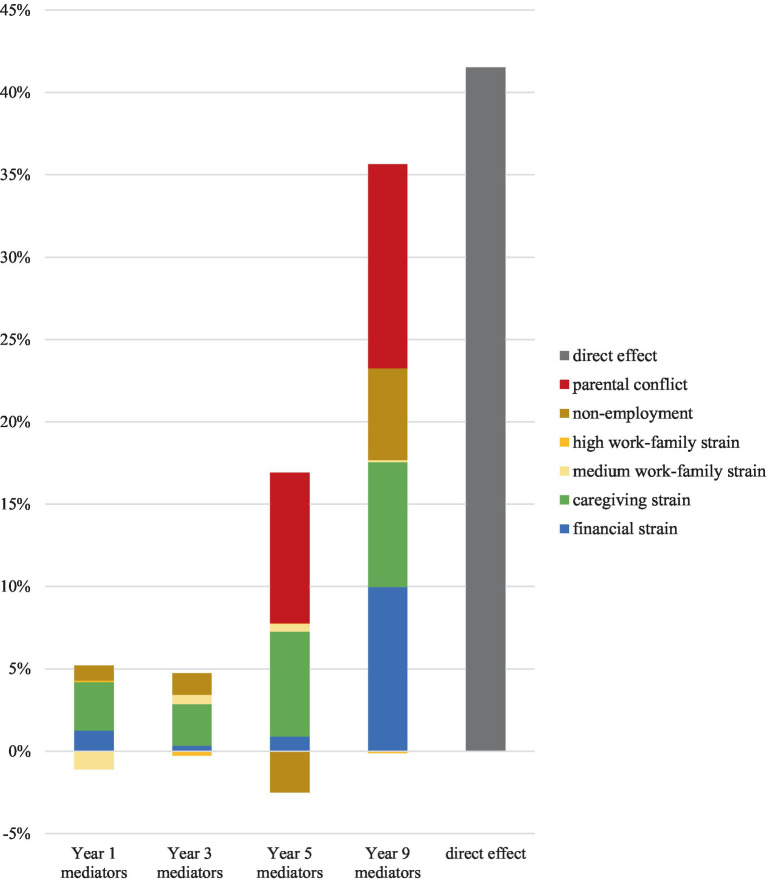
Proportion of difference in lone and partnered mothers’ depressive symptoms at Year 9 that is mediated by various current and past stressors (*n* = 5,654).

The model presented in Table 4 presents the contributions of each individual stressor to the combined mediation at Year 9. All current and past stressors were included in the mediation, but only mediators that contributed more than 1% of the indirect effect are reported in Table 4. These results are also displayed graphically in Figure 1. Additionally, in the Appendix (Table A1) we present the results for all mediators.

The results presented in Table 4 demonstrate that the timing of stress exposure matters for mothers’ mental health in Year 9. Although both current and past exposure to stressors contributed to the mediation, current stress exposure had the greatest impact, with stressors reported in Year 9 accounting for 61% of the indirect effect and 36% of the total effect. We observe that stressors reported in Year 5 accounted for 24% of the indirect effect, and 14% of the total effect. Meanwhile, stressors reported in Year 3 and Year 1 each accounted for 7% of the observed indirect effect and for 4% of the total effect each.

Table 4 also shows relevant differences in maternal depressive symptoms by family structure depending on the type of stressor examined. We can observe that parental conflict made the greatest contribution to the mediation among stressors reported in both Year 9 (12% of the total effect) and Year 5 (9%). No measure for parental conflict was available for Years 1 and 3, which impedes to quantify whether the effect of parental conflict on mothers’ mental health persists over longer stretches of time. Although current financial strain made a substantial contribution to the mediation (10% of the total effect), past financial strain made a relatively small contribution (1% or less), suggesting that stress related to money has a transitory impact on mothers’ mental health. Both current and past caregiving strain contributed to the mediation, though the size of the contribution was smaller for caregiving strain reported in Year 1 and Year 3 (3% of the total effect) than that reported in Year 5 (6%) and Year 9 (8%). Of the measures recording work-related strain, non-employment made the greatest contribution to the mediation. Interestingly, non-employment at Year 5 had a negative contribution to the mediation. This finding means that, once we controlled for all variables, non-employment in Year 5 had a buffering effect on maternal depressive symptoms, compared to working in a job with low-work family strain.

### 4.4 Robustness checks

In additional analyses, we applied different model specifications to capture the role of time-variant measures of family structure (i.e., transitioning from partnered to lone mother, and from lone to partnered mother) in explaining variations in maternal mental health. While our KHB decomposition analysis (Karlson et al., 2012) is suited to capture decomposition paths that require stable family structures across waves, our additional fixed-effects analyses as robustness checks permit to account for unobserved heterogeneity through examining changes across time points (Allison, 2009). These additional fixed-effects analyses offered results that are consistent with those from our random-effects models. First, when we restricted our analyses exclusively to families that were two-parent households in wave 1, we found that transitioning from partnered to lone mother is associated with increased depressive symptoms. Second, when restricting the analyses only to mothers who were lone-parent families in wave 1, we found that transitioning from lone to partnered motherhood is associated with reduced maternal depressive symptoms. These additional analyses give general support to the findings of our study, indicating solid statistical associations between family structure and mothers’ mental health after accounting for key demographic factors and for unobserved heterogeneity, which further justifies our mediation approach in testing the stress process model.

## 5 Discussion

This study has used longitudinal data from Ireland to examine how family structure shapes variations in mothers’ mental health, paying particular attention to the role of family stressors exposure. Most research on this topic has used small, cross-sectional samples. By contrast, we analysed four waves of high-quality data collected over a nine-year period for a large, nationally representative sample of mothers. We critically add to the existing family and health literatures by applying a dynamic mediation framework with multiple repeated measures of exposure to family stressors—i.e., financial strain, caregiving strain, work-related strain, and parental conflict—to investigate the relative role of stressors in explaining disparities in mental health (i.e., depressive symptoms) between lone and partnered mothers.

The findings of our study can be summarised at various levels. First, we found that lone mothers, compared to partnered mothers, are disadvantaged at multiple levels. Lone mothers reported around twice as many depressive symptoms as partnered mothers, and were also disadvantaged in their socioeconomic and demographic characteristics. Relative to partnered mothers, lone mothers entered parenthood at younger ages, had fewer years of education, and were more likely to report early economic deprivation in their families of origin. These findings echo existing research on the selective nature of lone motherhood (Hannan, 2018). Additionally, we show that lone mothers had a higher exposure to all stressors across different time points, compared to partnered mothers. Specifically, lone mothers reported more financial and caregiving strain, higher exposure to parental conflict, and a higher likelihood of being outside paid work and suffering from work–family conflict, relative to partnered mothers.

Second, our longitudinal multivariate analyses reveal that the higher risks of depressive symptoms among lone mothers persist after controlling for various sociodemographic factors. In the main random-effect models addressing within- and between-level variations, we found that, despite part of the association between family structure and mothers’ mental health is taken away when adding a number of sociodemographic controls in the analyses, a remarkable well-being disadvantage for lone mothers persists after these controls are added. Further, additional fixed-effects analyses conducted as robustness checks to address within-individual change in family structure status for various alternative sample specifications showed that (i) transitioning from being partnered to lone mother is associated with women’s higher depressive symptoms, and (ii) transitioning from lone to partnered mother is associated with reduced risks in women’s depressive symptoms. These findings indicate a solid and consistent result showing that lone mothers experience higher depressive symptoms than partnered mothers, net of sociodemographic factors.

Third, in our decomposition analyses, we found that multiple stressors contribute to explain the observed associations between family structure and maternal mental health. Although an important 41% of the statistical effect of family structure on mothers’ depressive symptoms was not explained by exposure to stressors, a larger 59% of this mental health gap was explained by the unequal exposure to stressors between lone and partnered mothers. We found that both current and past caregiving strain mediate the statistical effect of lone motherhood on maternal depressive symptoms after 9 years since childbirth. Current caregiving strain contributed to 8% of the total effect of lone motherhood on mothers’ depressive symptoms, whereas caregiving strain even in the year of childbirth explained 3% of the mediation. This finding indicates that the caregiving challenges of raising young children without a co-resident partner have lasting effects on lone mothers’ well-being. Also, current financial strain made a substantial 10% of the observed mediation, indicating that lone mothers’ financial instability plays an important role in explaining their mental health disadvantage. By contrast, financial strain reported at previous waves made only a small contribution to the mediation, accounting for 1% or less of the total effect. Regarding paid work, non-employment made the greatest contribution to the mediation, with the highest contribution observed for current non-employment, accounting for 6% of the mediation. Finally, both past and current parental conflict made relevant additions to explain gaps in depressive symptoms between lone and partnered mothers, with a contribution of 9% for parental conflict observed 5 years after childbirth, and up to 12% for current conflict. Overall, current caregiving strain, current and past parental conflict, as well as current financial strain, accounted for the largest share of the mediation explaining lone mothers’ mental health disadvantage.

Our findings have direct implications for the sociological literatures on family and health. Globally, our approach to study underlying mechanisms behind differences in mothers’ mental health across family structure reveals the importance of considering, not only different types of family stressors, but also a distinction between present and accumulated stressors over years. While the role of accumulated stress is at the core of the ‘stress process model’ (Amato, 2010; Pearlin, 1999), previous studies addressing differences in women’s mental health omitted such dynamic view on stress accumulation. Our study not only suggests that previous stress accumulation partly drives the inequalities in well-being between lone and partnered mothers. It also reveals the importance of specific stressors with different consequences for family relations and women’s well-being. Caregiving strains and parental conflict accumulate over the years to contribute to lone mothers’ disadvantage in mental health, whereas economic vulnerability matters too, but particularly so in the short term, rather than in the long term. These results resonate with previous research on the disadvantage of lone mothers linked to higher time poverty, energy demands and material constraints (e.g., Bernardi and Mortelmans, 2018; Nieuwenhuis and Maldonado, 2018). Additionally, these findings crucially reveal how such structural disadvantages lead to vital processes of stress accumulation that subsequently influence lone mothers’ poorer mental well-being.

The results of this paper must be discussed by considering its context of study: Ireland. Previous scholarship on welfare, family, and gender regimes indicates that Ireland combines limited universal policy support oriented to families with dependent children, including lone-parent families, with a clear persistence of gender traditional roles and expectations, despite signs of social changes in family relations over the last decades (Canavan, 2012; Daly, 2018; McGinnity and Russell, 2008). Previous research found that Ireland has a very high parenthood “happiness penalty,” using a cross-country approach (Glass et al., 2016). Our longitudinal study goes beyond previous Irish cross-sectional research (Fahey et al., 2012) by suggesting that this well-being penalty is particularly strong among lone mothers, as shown by their high incidence of depressive symptoms. While conservative values of social stigma could act as mechanisms driving mental health gaps between lone and partnered mothers in Ireland, our study indicates that these gaps could be reduced with stronger policy support to lone-mother families in Ireland. Based on our results, family polices targeting poverty risks among single-parent families and more extensive parental support to balance paid and unpaid work in lone-parent homes in Ireland (e.g., flexible paid work conditions, lower child care costs) may contribute to reduce critical stressors among lone mothers, which are found to be associated with their high incidence of mental health problems. Future studies should further examine how micro-level and macro-level contexts interact to shape inequalities in parental mental health by family structure. To achieve this goal, cross-national comparisons across countries with different policy and cultural characteristics will be needed.

This study presents some limitations that must be acknowledged. First, our dependent variable was limited to mothers’ depressive symptoms, as unfortunately other mental health measures (e.g., anxiety) were not available in the GUI data. While depressive symptoms is highly correlated to other mental health outcomes, future research on different well-being outcomes is needed. Second, we did not have space to focus on buffers, which are essential to the ‘stress process model’ (Pearlin, 1999). Future research should examine how certain types of resources, such as social support and maternal self-concept, may buffer lone mothers against the unequal impact of their exposure to stressors. Third, we were unable to study fathers, due to sample size limitations. Lone fathers are an understudied group that requires future attention in this field, considering the gendered processes affecting parental well-being outcomes in lone-parent and two-parent families (Chiu et al., 2017; Costanzo et al., 2024; Roeters and Gracia, 2016; Wade et al., 2011). Fourth, for reasons of space and modelling strategy, our mediation framework was restricted to stable groups of family structure. Although our decomposition approach was able to capture key stress-related mechanisms behind mental health gaps between lone and partnered mothers, future research should examine how different types of family structures—stable and changing—are associated with disparities in mothers’ mental health outcomes. Further research may contribute to the growing sociological literature addressing disparities in women’s economic and mental well-being across family structure over the life course (Kreyenfeld and Trappe, 2020; Kühn et al., 2023; Struffolino et al., 2016).

To conclude, despite some shortcomings, this study adds to previous literature by illustrating how previous and current family stressors contribute to explain disparities in mental health between lone and partnered mothers. New research using diverse methods (e.g., family interventions, lab experiment designs, in-depth qualitative interviews) should further add to capture underlying mechanisms linking exposure to stressors to differences in parental mental health by family structure. Our empirical approach, we hope, will add new insights to guide future sociological research addressing inequalities in parental well-being across different family structures.

## Data Availability

The data analyzed in this study is subject to the following licenses/restrictions: the data used come from the Growing Up in Ireland (GUI). GUI was funded and managed by the DCEDIY in association with the Central Statistics Office (CSO) and only available for restricted purposes. Results in this scientific article are based on analyses of data from Research Microdata Files provided by the CSO. Neither the CSO nor DCEDIY take any responsibility for the views expressed or the outputs generated from these analyses. Requests to access these datasets should be directed to https://www.growingup.gov.ie/.

## Appendix

**Table A1:** Components of difference from the decomposition of the effect of family structure on mothers’ depressive symptoms at Year 9 using the KHB method (*n* = 5,654).

Z-variable	*Est.*	*S.E.*	% indirect effect	% total effect
*Year 9 mediators*				
Financial strain	0.16	0.06	17%	10%
Caregiving strain	0.12	0.06	13%	8%
Medium work-family strain^a^	0.00	0.01	0%	0%
High work-family strain^a^	0.00	0.01	0%	0%
Non-employment^a^	0.09	0.04	10%	6%
Parental conflict	0.20	0.07	21%	12%
*Year 5 mediators*				
Financial strain	0.01	0.03	2%	1%
Caregiving strain	0.10	0.05	11%	6%
Medium work-family strain^a^	0.01	0.01	1%	0%
High work-family strain^a^	0.00	0.01	0%	0%
Non-employment^a^	−0.04	0.03	−4%	−3%
Parental conflict	0.15	0.07	16%	9%
*Year 3 mediators*				
Financial strain	0.01	0.03	1%	0%
Caregiving strain	0.04	0.03	4%	3%
Medium work-family strain^a^	0.01	0.01	1%	1%
High work-family strain^a^	0.00	0.01	0%	0%
Non-employment^a^	0.02	0.02	2%	1%
*Year 1 mediators*				
Financial strain	0.02	0.03	2%	1%
Caregiving strain	0.05	0.03	5%	3%
Medium work-family strain^a^	−0.02	0.01	−2%	−1%
High work-family strain^a^	0.00	0.03	0%	0%
Non-employment^a^	0.01	0.02	2%	1%

a Reference category is employed with low work-family strain.
